# Mapping cardiac microstructure of rabbit heart in different mechanical states by high resolution diffusion tensor imaging: A proof-of-principle study

**DOI:** 10.1016/j.pbiomolbio.2016.06.001

**Published:** 2016-07

**Authors:** Irvin Teh, Rebecca A.B. Burton, Darryl McClymont, Rebecca A. Capel, Daniel Aston, Peter Kohl, Jürgen E. Schneider

**Affiliations:** a Division of Cardiovascular Medicine, Radcliffe Department of Medicine, University of Oxford, Oxford, United Kingdom; b Department of Pharmacology, University of Oxford, Oxford, United Kingdom; c National Heart and Lung Institute, Imperial College London, London, United Kingdom; d Institute for Experimental Cardiovascular Medicine, University Heart Centre Freiburg - Bad Krozingen, Medical School of the University of Freiburg, Germany

**Keywords:** Rabbit heart, Myocardial tissue characterisation, Diffusion tensor imaging, Tractography, Diastole, Systole

## Abstract

Myocardial microstructure and its macroscopic materialisation are fundamental to the function of the heart. Despite this importance, characterisation of cellular features at the organ level remains challenging, and a unifying description of the structure of the heart is still outstanding. Here, we optimised diffusion tensor imaging data to acquire high quality data in *ex vivo* rabbit hearts in slack and contractured states, approximating diastolic and systolic conditions. The data were analysed with a suite of methods that focused on different aspects of the myocardium. In the slack heart, we observed a similar transmural gradient in helix angle of the primary eigenvector of up to 23.6°/mm in the left ventricle and 24.2°/mm in the right ventricle. In the contractured heart, the same transmural gradient remained largely linear, but was offset by up to +49.9° in the left ventricle. In the right ventricle, there was an increase in the transmural gradient to 31.2°/mm and an offset of up to +39.0°. The application of tractography based on each eigenvector enabled visualisation of streamlines that depict cardiomyocyte and sheetlet organisation over large distances. We observed multiple V- and N-shaped sheetlet arrangements throughout the myocardium, and insertion of sheetlets at the intersection of the left and right ventricle. This study integrates several complementary techniques to visualise and quantify the heart’s microstructure, projecting parameter representations across different length scales. This represents a step towards a more comprehensive characterisation of myocardial microstructure at the whole organ level.

## 1 Introduction

The heart’s pump function depends on cardiomyocyte activity. These cells occupy the majority of the ventricular volume, and are organised in a highly structured three-dimensional (3D) pattern (Kohl, 2003). The two perhaps most prominent macroscopic descriptors are (i) locally prevailing cardiomyocyte orientation, often referred to as ‘fibre orientation’ (Holmes et al., 2000), and (ii) laminar structures of laterally interconnected myocyte layers known as ’sheetlets’ (Hales et al., 2012, Sands et al., 2005). The organisation and precise distribution of cardiac cells play important roles in the complex electromechanical properties in the heart (Katz and Katz, 1989). During systole, a combination of cardiomyocyte contraction and shear between layers of cardiomyocytes leads to longitudinal shortening of the ventricle in the form of an atrio-ventricular valve plane shift, radial thickening of the ventricle wall predominantly in a centripetal direction, and torsion of the ventricles by apical rotation relative to the base of the heart (Axel et al., 2014). The 3D organisation of fibres and sheetlets has been shown to influence both mechanical contraction (Waldman et al., 1988) and electrical propagation (Kanai and Salama, 1995) in the heart, and has been implicated in ventricular hypertrophy (Ferreira et al., 2014, McGill et al., 2012, Tseng et al., 2006), infarct remodelling (Wickline et al., 1992) and arrhythmia (Chen et al., 1993). Accurate characterisation of the myocardial architecture is therefore fundamental for understanding its role in health and disease.

Traditional histo-anatomical characterisation methods, such as gross anatomical dissection or histology, have laid the foundation for our current understanding of cardiac structure (Streeter Jr, 1979). While the presence of helical fibre alignment in the heart has been known for centuries (Lower, 1669), more recent studies have shown that fibre orientation changes from a left-handed helix at the epicardium to a right-handed helix in the endocardium (Streeter et al., 1969). Due to excellent resolution and cell-type specificity, histological methods form a ‘gold standard’ for tissue characterisation. At the same time, these techniques are associated with tissue distortion and disruption during sample preparation, limited spatial coverage, and non-trivial challenges during reconstruction of whole heart two-dimensional (2D) data stacks into 3D volumes (Burton et al., 2014, Plank et al., 2009).

Other tissue imaging methods are available. These include anatomical magnetic resonance imaging (MRI) (Bernus et al., 2015, Burton et al., 2006) and X-ray computed tomography (Ni et al., 2013), with excellent sample coverage suitable for whole organ studies, but at a resolution in the 10^−5^ m region, they do not yet resolve cellular features. Polarised light imaging also offers good coverage, but resolution is limited to the 10^−5^ m range, and fibre orientation estimates are sensitive to the elevation angle of the cells (Jouk et al., 2007). Scanning electron microscopy (LeGrice et al., 1995) and confocal microscopy (Young et al., 1998), in contrast, provide the highest resolution of 10^−9^ m and better, but are limited in field-of-view.

Diffusion tensor imaging (DTI) (Basser et al., 1994) enables 3D structural mapping of whole hearts. While the imaging resolution is on the order of 10^−4^ m, the technique is sensitive to the microscopic diffusion of water in the 10^−6^ m range, and its interactions with the cellular environment. This facilitates assessment of the 3D arrangement of cardiac fibres and sheetlets (Helm et al., 2005, Scollan et al., 1998). In DTI, the diffusion of water is modelled as a 3D tensor. The lengths and orientations of the three mutually orthogonal major axes (longest, intermediate and shortest) of this tensor are described respectively by its eigenvalues and eigenvectors. The primary, secondary and tertiary eigenvectors (**v**_**1**_, **v**_**2**_, **v**_**3**_) are understood to correspond to the fibre long-axis, the fibre-normal/in-sheet and the sheetlet-normal directions respectively, of cells in myocardial laminae (Helm et al., 2005, Scollan et al., 1998), as validated by histology (Holmes et al., 2000, Hsu et al., 1998, Kung et al., 2011, Scollan et al., 1998).

Several studies have utilised DTI to quantitatively investigate rabbit heart structure. Early work characterised fibre and sheetlet orientations in healthy rabbit hearts, and linked **v**_**3**_ to the orientation of the ventricular sheetlets (Scollan et al., 2000). Rabbit hearts have also been used in important histological validation studies of DTI, which clarified the correspondence of the diffusion signal to the underlying microstructure (Holmes et al., 2000, Scollan et al., 1998). In these studies, 2D fast spin echo DTI data were acquired at an imaging resolution of 156 × 312 × 2000 μm. More recent work investigated how well the fibre orientation is conserved across species in mice, rabbit and sheep (Healy et al., 2011). Using a 3D spin echo acquisition at 250 μm isotropic resolution, the study focused only on the helix angle of **v**_**1**_ in the left ventricle.

Improvements in magnet and gradient system hardware, as well as data acquisition protocols, now allow one to acquire high quality DTI data at comparatively high isotropic resolution in the isolated fixed heart. Here, we combine a state-of-the-art preclinical MRI system with optimised 3D spin echo DTI acquisition to improve the signal-to-noise ratio, supporting an isotropic imaging resolution of 200 μm in *ex vivo* rabbit hearts. Both **v**_**1**_ and **v**_**3**_ are assessed and quantified in multiple wall regions and heights in both the left ventricle (LV) and right ventricle (RV). Superquadric glyphs are used to aid voxel-wise display of tensors, transmural angle profiles are quantified regionally, and global tractography is employed to help visualise macroscopic distributions of fibres and sheetlet alignments. The study (i) uses multiple approaches to augment our understanding of myocardial structure in rabbit hearts, and (ii) investigates structural differences between the diastolic (slack) and systolic (contracture) states.

## 2 Materials and methods

### 2.1 Sample preparation

All experiments were conducted in accordance with the Animals (Scientific Procedures) Act 1986 (UK). Hearts were isolated from two male New Zealand White rabbits after induction of terminal anaesthesia (pentobarbitone under Schedule 1 protocol). Isolated hearts were swiftly cannulated and perfused in constant pressure Langendorff mode for 5 min with normal physiological saline (in mM: NaCl 125, NaHCO_3_ 25, KCl 5.4, NaH_2_PO_4_ 1.2, MgCl_2_ 1, Glucose 5.5, CaCl_2_ 1.8, pH to 7.4 with NaOH and oxygenated with 95% O2/5% CO2) containing heparin (5 IU/mL). The first heart was arrested using physiological saline with elevated potassium (20 mM KCl) to induce diastolic arrest, while the second heart was exposed to lithium-replacement of sodium to induce contracture (in mM: LiCl 125, KCl 5, MgCl_2_ 1, HEPES 10, Glucose 11, CaCl_2_ 2.5, pH 7.4) (Burton et al., 2006). Hearts were then perfused via the aorta with isosmotic Karnovsky’s fixative (300 ± 10 mOsm, Solmedia UK) while immersed in fixative to avoid ingress of air into cardiac chambers. Following a fixation period of 4 days, hearts were placed in fixative containing 2 mM gadolinium (Gd) complex Prohance (Bracco, MN, USA). Prior to imaging, the hearts were washed in PBS + 2 mM Gd, and embedded in 1% low melting point agarose gel made from PBS + 2 mM Gd.

### 2.2 Data acquisition

Non-selective 3D spin echo DTI data were acquired on a 9.4 T preclinical MRI scanner (Agilent, CA, USA) with a shielded gradient system (max gradient strength = 1 T/m, rise time = 130 μs), and transmit/receive quadrature coil (inner diameter = 42 mm; Rapid Biomedical, Rimpar, Germany). Acquisition parameters were: repetition time = 250 m, echo time = 10 m, field-of-view = 43.2 × 28.8 × 28.8 mm, matrix size = 216 × 144 × 144, resolution = 200 × 200 × 200 μm, number of non-diffusion-weighted (DW) images = 3, number of DW directions = 12 (Cook et al., 2007), diffusion duration (δ) = 2 m, diffusion time (Δ) = 5.5 m, b-value = 1000 s/mm^2^, acquisition time = 21:36 h. Based on these diffusion times and assuming unrestricted diffusion, the root mean squared displacement in tissue with apparent diffusivities ranging from 1.0–1.5 × 10^−3^ mm^2^/s would have ranged from 3.1 to 3.8 μm (Latt et al., 2007). Sample temperature variations, arising from warming of the gradient coils during the application of diffusion gradients, were minimised by interleaving DW and non-DW scans. The receiver gain was increased by 10 dB during the DW scans to increase the signal-to-noise ratio, while remaining within the dynamic range of the receiver. The amplified DW signals as a result of the higher gain were normalised by separate non-localised gradient echo data acquired at the respective receiver gains. The gradient system was calibrated prior to the experiment to minimise directional bias in the diffusion measurements (Teh et al., 2016).

### 2.3 Data analysis

3D tensors were fitted to the DTI data using non-linear least squares. Manual segmentation of the LV and RV myocardium was performed based on reconstructed fractional anisotropy (FA) maps (Pierpaoli and Basser, 1996). The DTI data were then registered as follows: centroids of the LV cavity in each axial slice were detected automatically and a line, defining the long axis, was fit to the centroids. The DTI data were aligned to the long-axis, and the LV-RV intersections at the anterior and posterior walls in a mid-myocardial short-axis slice were manually specified. The interconnecting line between the two points was defined as the anterior-posterior direction. The DTI data were then rotated in-plane to the new reference frame. The b-matrix was correspondingly transformed in 3D to match the rotated images (Leemans and Jones, 2009). Diffusion tensors were then recomputed based on the registered data.

**Fig. 1:**
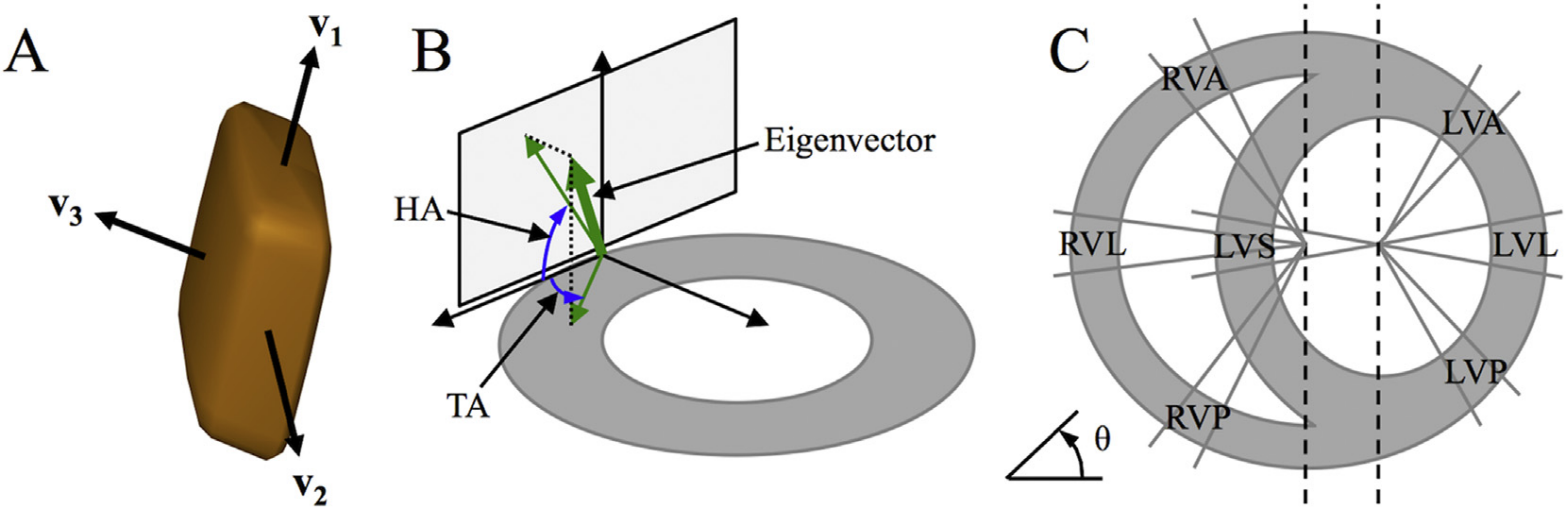
Definition of eigenvectors, angles, and segments. A: Single tensor, displayed as a superquadric glyph, shows the orientation of the principal eigenvectors **v**_**1**_, **v**_**2**_ and **v**_**3**_. B: The helix angle (HA) of a given eigenvector is the angle subtended by the projection of the eigenvector onto the tangential plane and the short-axis plane. The transverse angle (TA) is the angle subtended by the projection of the eigenvector onto the short-axis plane and the tangential plane. C: Specification of regions-of-interest in the LV anterior, lateral, posterior and septal walls (LVA, LVL, LVP and LVS) based on the angle θ, perpendicular to the long-axis centred in the LV cavity. The RV anterior, lateral and posterior walls (RVA, RVL, RVP) were similarly specified based on a long-axis centred on the union of the LV septum and cavity and the RV cavity.

Parametric maps, including the mean apparent diffusion coefficient (ADC), principal eigenvalues λ_1_, λ_2_ and λ_3_, FA and transverse anisotropy λ_2_/λ_3_, were calculated (Pierpaoli and Basser, 1996) and reported for the LV and RV in slack and contractured states. To quantify precision in **v**_**1**_, **v**_**2**_ and **v**_**3**_, wild bootstrapping was performed (Whitcher et al., 2008), and 95% cone-of-uncertainty (COU) are reported. Masks based on the signal intensity of the B = 0 image, mean ADC and FA were used to exclude the surrounding gel and buffer within the ventricles. Superquadric shape functions describe a wide range of shapes from spheres to discs and cubes, and are better suited in highlighting the orientations of **v**_**2**_ and **v**_**3**_ than traditional ellipsoids (Ennis et al., 2005). As illustrated in Fig. 1, tensors were displayed as superquadric glyphs for improved visualisation of myocardial sheetlet arrangements. Tracking was performed in the whole heart along **v**_**1**_ and **v**_**3**_ using Diffusion Toolkit (Wang et al., 2007). The *fibre assignment by continuous tracking* algorithm (Mori et al., 1999) was used, along with a spline filter and an angle threshold of 30°. A threshold of FA ≥ 0.11 was used to segment the heart, and tracks were seeded from all voxels within the segmented volume. The tracks in the whole heart and intersecting various regions-of-interest (ROIs) were displayed in Trackvis (Wang et al., 2007). The term ‘fibres’ is used to describe the locally prevailing cell orientations as assessed by **v**_**1**_ in DTI. ‘Tracks’ refer to the streamlines connecting contiguous tensors with similar alignment. A minimum length threshold of 0.5 mm was used for **v**_**1**_ tracks. **v**_**1**_ and **v**_**3**_ tracks were colour-coded by orientation: apico-basal (red), anterior-posterior (green) and lateral-septal (blue).

In order to mitigate variations arising from local tissue deformations, the helix angles (HA) and transverse angles (TA) were defined relative to a local coordinate system. Local radial unit vectors were computed using Laplace’s method in the LV and RV separately (Jones et al., 2000). Local circumferential unit vectors were defined as being perpendicular to the local radial and global longitudinal unit vectors. Finally, the local longitudinal unit vectors were derived from the local radial and circumferential unit vectors. Short-axis planes were defined by the local radial and circumferential unit vectors. Tangential planes were defined by local longitudinal and circumferential unit vectors.

Typically, HA and TA reflect the orientation of **v**_**1**_ in the long-axis and its deviation from the circumferential axis in the short-axis plane respectively. In order to assess sheetlet structures, we similarly define HA and TA based on **v**_**3**_. The HA of a given eigenvector is the angle subtended by the projection of the eigenvector onto the tangential plane and the short-axis plane. The TA is the angle subtended by the projection of the eigenvector onto the short-axis plane and the tangential plan. HA (**v**_**1**_, **v**_**3**_) and TA (**v**_**1**_, **v**_**3**_) maps were generated in the whole heart. A custom cyclic colour map was used to avoid discontinuities at transitions between −90° and +90°.

ROIs in the LV and RV myocardium were identified according to Fig. 1. Segments of LV myocardium corresponding to angular projections (θ) in the short-axis plane were defined as the LV lateral (−10° < θ < 10°), anterior (40° < θ < 60°), septal (170° < θ < 190°) and posterior (300° < θ < 320°) walls (Healy et al., 2011). Similarly, segments in the RV myocardium were defined as the RV anterior (120° < θ < 140°), lateral (170° < θ < 190°) and posterior (220° < θ < 240°) walls. The transmural profiles of HA (**v**_1_, **v**_3_) and TA (**v**_1_, **v**_3_) were reported in these ROIs in a basal, middle and apical slice. To do so, HA and TA, corresponding to each ROI, slice and heart, were averaged in polar coordinates within radial bins of 0.2 mm. HA and TA were then reported as a function of myocardial wall thickness. Wrap-around of HA and TA from −90° to +90° and vice versa, as occurring particularly in epicardial and endocardial regions, was avoided by expanding the dynamic angle range to −150° to +150°. The linearity of the profiles was characterised by the R^2^ obtained with a linear fit (Healy et al., 2011). The total angle gradients over the myocardial walls are reported in °/mm. Histograms of HA (**v**_**1**_, **v**_**3**_) and TA (**v**_**1**_, **v**_**3**_) were computed in the LV and RV across three adjacent mid-ventricular slices with a bin size of 2°. All processing was performed using in-house code (Matlab2013a, Natick, MA, USA).

## 3 Results

**Fig. 2:**
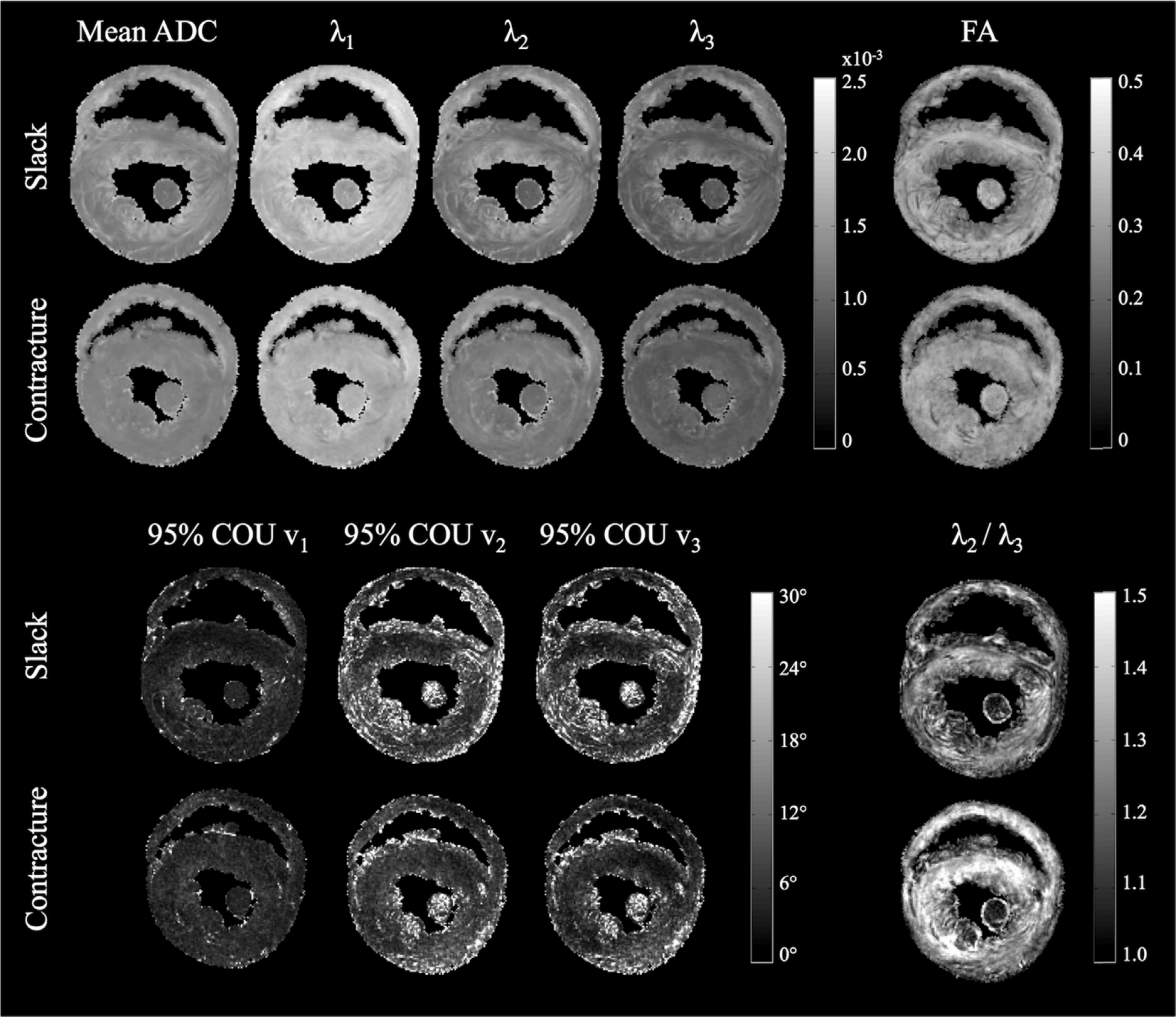
Parametric maps in a mid-myocardial short-axis slice. (Top from left to right): Mean apparent diffusion coefficient (ADC), principal eigenvalues λ_1_, λ_2_, and λ_3_, and fractional anisotropy (FA). (Bottom from left to right): 95% cone-of-uncertainty (COU) in **v**_**1**_, **v**_**2**_ and **v**_**3**_, and transverse anisotropy (λ_2_/λ_3_).

Diffusion parameter maps in a mid-ventricular slice in slack and contractured hearts are presented in Fig. 2. λ_1_ was relatively homogeneous across the myocardium in the slack heart, whereas λ_2_ and λ_3_ were elevated in the subendo- and subepicardium, contributing to a locally higher mean ADC and lower fractional and transverse anisotropy. Data from the contractured heart were less heterogeneous than in the slack heart. Transverse anisotropy was higher is the contractured heart, but remained low within the papillary muscles. Summary data of these observations in the whole heart are presented in Table 1. Mean ADC and transverse anisotropy were 6.7% lower and 4.9% higher respectively, in the LV of the contractured heart compared to the LV of the slack heart. Similarly, the same values in the RV of the contractured heart were 3.5% lower and 3.2% higher than those of the slack heart respectively. Average FA was unchanged. Correspondingly, λ_1_, λ_2_ and λ_3_ were lower in both the LV and RV of the contractured heart, by up to 8.8%.

In terms of measurement precision, 95% COU (**v**_**1**_/**v**_**2**_/**v**_**3**_) = 6.2°/14.8°/13.6° (slack) and 6.7°/12.8°/11.3° (contracture) averaged across the myocardium. Maps of 95% COU show that precision of **v**_**1,**_ **v**_**2**_ and **v**_**3**_ are excellent throughout most of the myocardium, and poorer where there are major vessels and edges with residual partial volume. In addition, precision of **v**_**2**_ and **v**_**3**_ are also poorer in the papillary muscles, in myocardial regions containing greater extracellular space and in regions near the apex. We find that 99%/90%/91% (slack) and 99%/93%/94% (contracture) of voxels in the myocardium fall within a threshold of 95% COU (**v**_**1**_/**v**_**2**_/**v**_**3**_) < 30°.

**Fig. 3:**
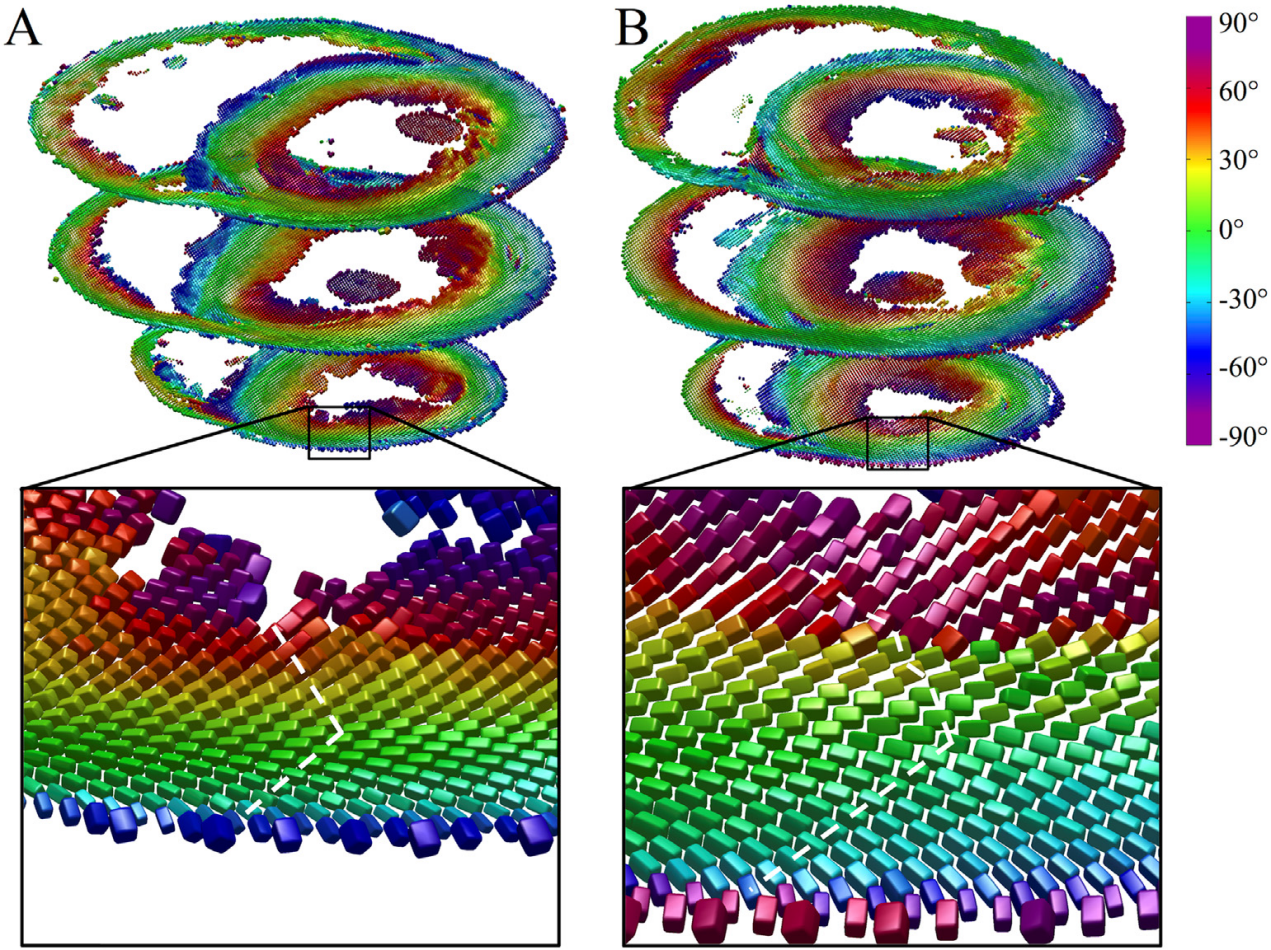
Diffusion tensors in apical, middle and basal short-axis slices in A: slack and B: contractured hearts. The tensors are coloured by helix angle of **v**_**1**_ and represented as superquadric glyphs to highlight the orientations of **v**_**2**_ and **v**_**3**_. A closer view of the LV anterior wall in an apical slice shows the extent of wall thickening in the contractured heart. Illustrative profiles tracking **v**_**3**_ (white dashed lines) shows a regular V-shaped sheetlet structure, which is less well-aligned in the contractured heart.

Fig. 3 illustrates tensors in three short-axis slices as superquadric glyphs, coloured by the helix angle of **v**_**1**_. Besides the orientation of **v**_**1**_, these glyphs highlight the orientations of **v**_**2**_ and **v**_**3**_ more clearly than ellipsoids. A close up of tensors in the slack heart illustrates the known circumferential arrangement of **v**_**1**_ and the transmural variation in helix angle, from negative in the subepicardium to positive in the subendocardium. **v**_**3**_ was oriented radially at the midline, but fanned out towards the edges of the heart giving a characteristic V-shaped transmural sheetlet profile. This V-shaped sheetlet arrangement was less coherent in the contractured heart between the subendocardium and midline.

**Fig. 4:**
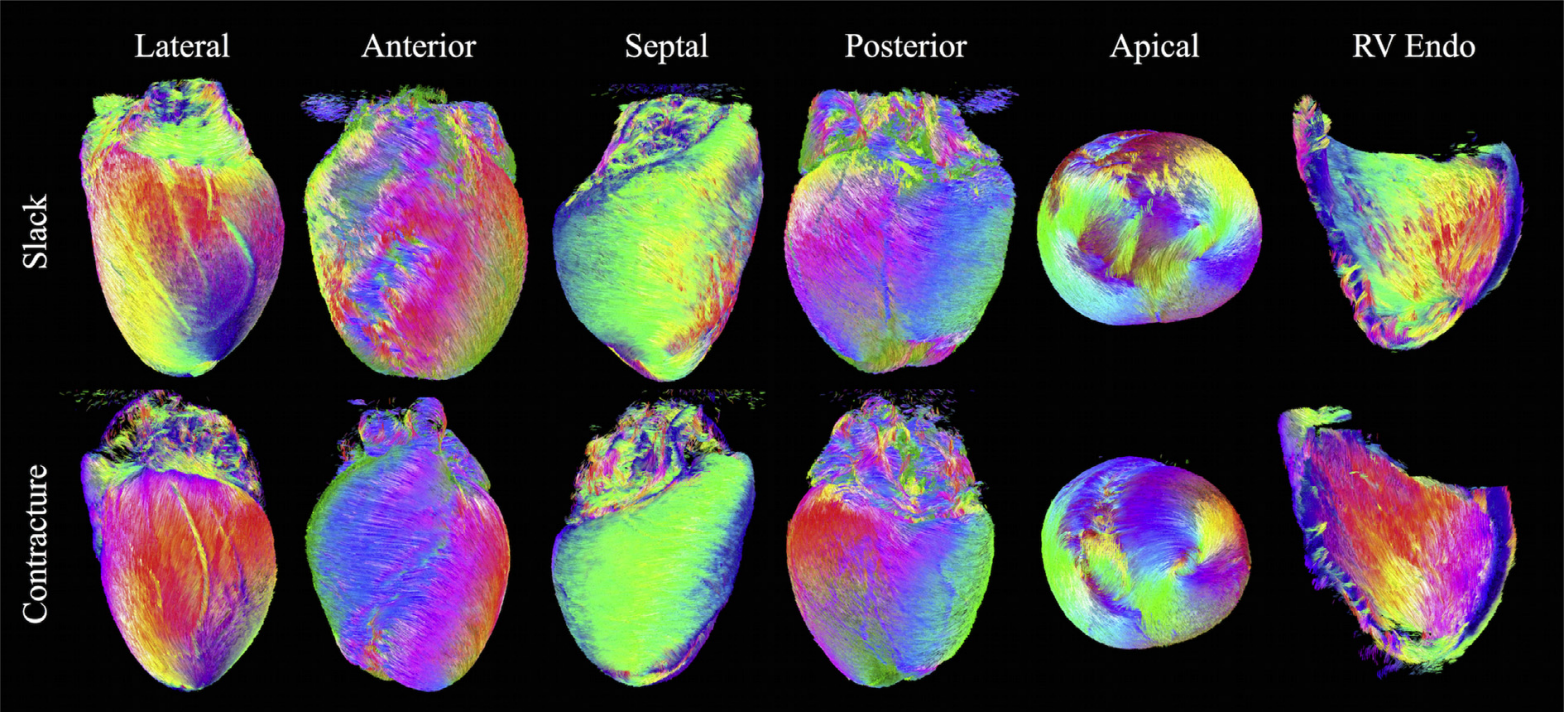
Tracking in slack and contractured hearts based on connecting **v**_**1**_ tensors. Left to right: Lateral, anterior, septal, posterior, and apical views of the epicardial surface. Tracks were colour-coded by orientation: apico-basal (red), anterior-posterior (green) and lateral-septal (blue).

Multiple epicardial views of tracks in the two hearts are shown in Fig. 4, along with an endocardial view of the RV wall. In the LV lateral wall of the slack heart, a thin outer layer of tracks is aligned longitudinally over a larger region of obliquely oriented left helical tracks. Tracks continue around the anterior surface of the heart where they interdigitate in bundles with left helical tracks of the RV. From the septal view, these tracks of the RV take on a more circumferential orientation. Tracks then revert to a left helical orientation at the posterior wall and blend in seamlessly with the epicardial tracks of the LV. In the apical view, tracks of the LV spiral clockwise towards the LV apex. In contrast, the RV apex is composed primarily of a combination of septally oriented tracks radiating from the LV apex, and longitudinally oriented tracks extending from the RV endocardium. Tracks in the RV endocardium can be seen to be generally right handed, with steeper helix angles near the LV posterior wall. In the contractured heart, tracks at the LV lateral wall epicardium are oriented more longitudinally than in the slack heart. Tracks in the RV epicardium, seen from anterior and septal aspects, are more circumferential. They interdigitate more pronouncedly with LV epicardial tracks in the anterior view, forming a suture-like feature in the apical view. We observe that LV tracks in the contractured heart spiral more uniformly towards the LV apex. RV endocardial tracks are again right-handed but with steeper helix angles. Besides the LV and RV, tracks forming the left and right atria, left atrial appendage, pulmonary artery and aorta can be seen. Gaps in the tracks in the LV wall correspond to major coronary vessels.

**Fig. 5:**
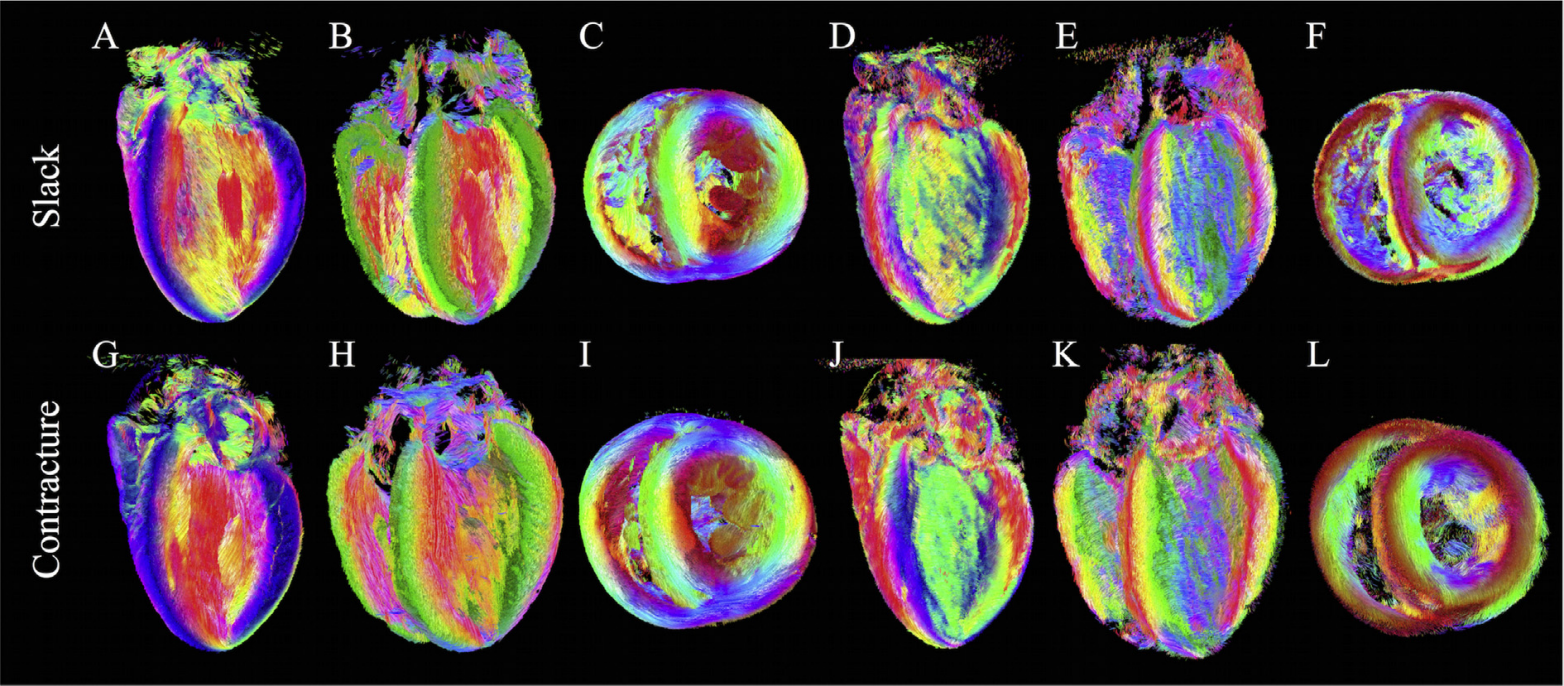
Tracking of fibre and sheetlet structures in digitally bisected hearts. A to C: Lateral, anterior and apical views of a slack heart with tracking based on **v**_**1**_, D to F: **v**_**3**_. G to L: Corresponding views in the contractured heart.

Digital bisections of the heart in the vertical long-axis, horizontal long-axis and short-axis are presented in Fig. 5. Tracking based on both **v**_**1**_ and **v**_**3**_ is shown. The **v**_**1**_ data in the slack heart (Fig. 5A–C) recapitulate the transmural transition in helix angle fairly consistently across a mid-myocardial short-axis slice of the LV. This left to right helical transition from the subepicardium to subendocardium is replicated in the RV. Papillary muscles and chordae tendinae are predominantly longitudinal in orientation. Subtle differences in track orientation can be seen throughout the contractured heart (Fig. 5G–I). A key difference is the marked decrease in HA in the LV lateral wall and increase in **v**_**1**_ HA in the LV septal wall, such that the majority of tracks in these segments were left-handed and right-handed respectively. Tracks based on **v**_**3**_ are more discontinuous than those based on **v**_**1**_ as they follow the nominal sheetlet-normal direction. **v**_**3**_ tracks also tend to form more discrete segments, with sharper orientation differences between adjacent segments (Fig. 5D–F). This gives rise to V- and N-shaped transmural sheetlet-normal profiles which occur throughout the LV wall, and are particularly evident in the long-axis views. V-shaped **v**_**3**_ distributions can also be seen in the RV wall. At the bottom of the short-axis view, longitudinally oriented **v**_**3**_ tracks of the RV can be seen to insert into the more radially oriented tracks of the LV. This contrast is not visible by tracking **v**_**1**_ (Fig. 5C). All features described are also seen in the contractured heart (Fig. 5J–L), with differences in the specific angle distributions.

**Fig. 6:**
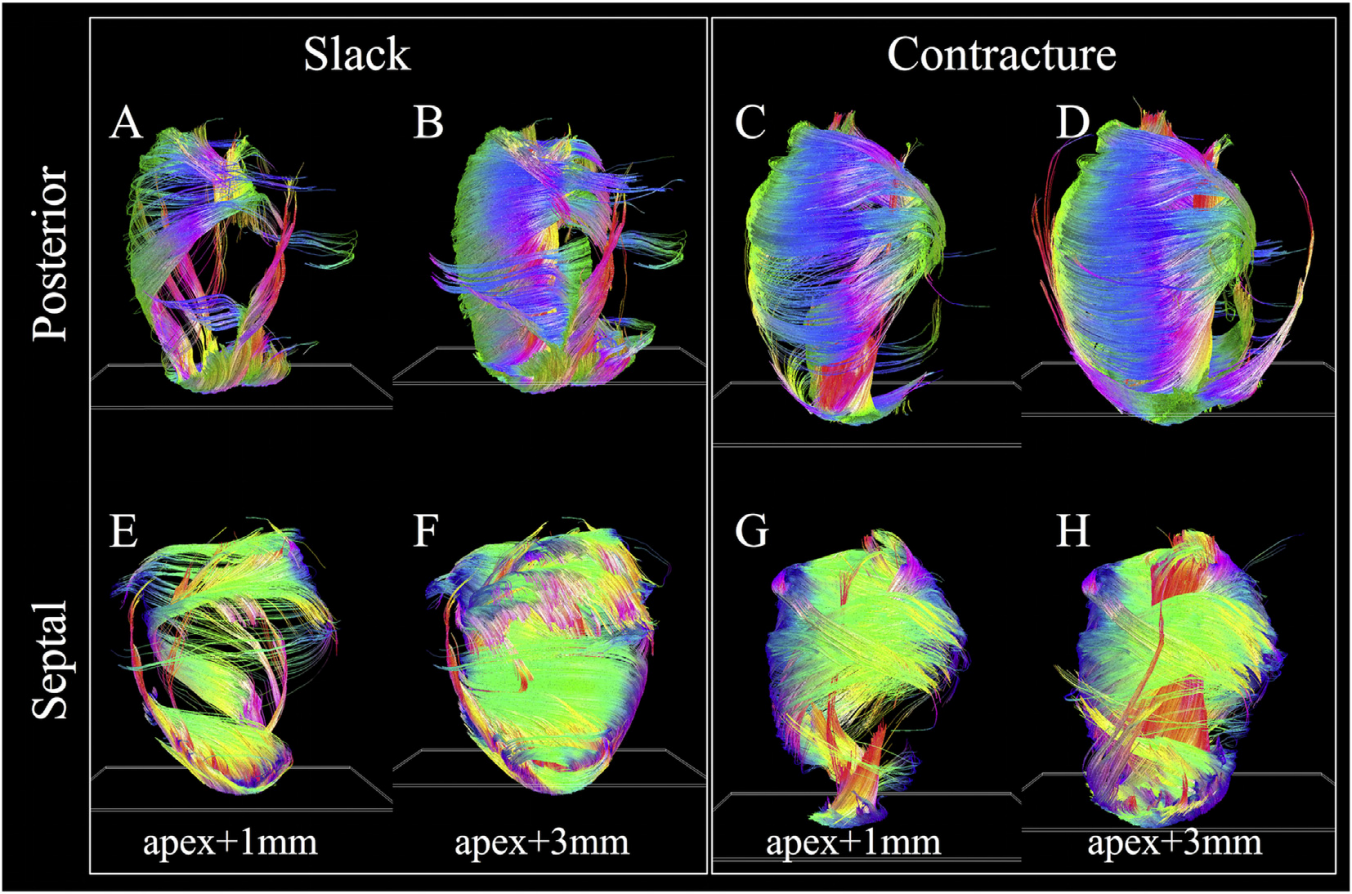
**v**_**1**_ tracks intersecting with single-voxel thickness short-axis planes (white oblique rectangles near the apex) offset by 1 mm and 3 mm from the apex. Posterior and septal views are presented for clarity.

Fig. 6 highlights the substantial differences in distribution of **v**_**1**_ tracks in hearts in slack and contracture. The tracking parameters and short-axis plane seed regions were the same to avoid introducing operator bias. In the slack heart (Fig. 6A–B; E–F), several groups of tracks were seen to arise from the apex. These include longitudinal tracks in the LV lateral subendocardium, some of which wind around towards the base of the heart in a right helical manner, left helical tracks of the LV septal endocardium and longitudinal tracks of the LV septal epicardium. As the specified seed plane is shifted from apex +1 mm to apex +3 mm, more tracks follow the above trajectories, forming much of the circumferential tracks of the LV lateral wall. In addition, longitudinal tracks were seen to rise up the LV posterior subendocardium. These wind in a right helical manner to form the LV septal epicardium. Tracks are also seen to wind in a left helix from the LV lateral epicardium, up the anterior wall, to form the circumferential tracks of the LV septal and posterior walls between the apex and mid-myocardium. In the contractured heart (Fig. 6C–D; G–H), major differences are observed. Starting from the apex, a thick band of right helical tracks is seen to arise from the LV lateral apical wall, moving up the LV subendocardium and LV posterior wall, before transitioning to circumferential tracks of the LV mid-myocardial septal wall, then to the left helical tracks of the LV basal anterior and lateral subepicardium and circumferential tracks of the LV posterior wall. With the seed plane shifted to apex +3 mm, another major band of tracks extend from the LV posterior apical subendocardium to the LV septal basal subendocardium in a steep right helix. These two bands of tracks combine to form a laminated fan-like array of tracks that meet at the LV septal basal wall. Separately, longitudinal tracks are seen to extend right helical-wise from the LV posterior subendocardium to the LV septal epicardium in a similar manner as in the slack heart.

**Fig. 7:**
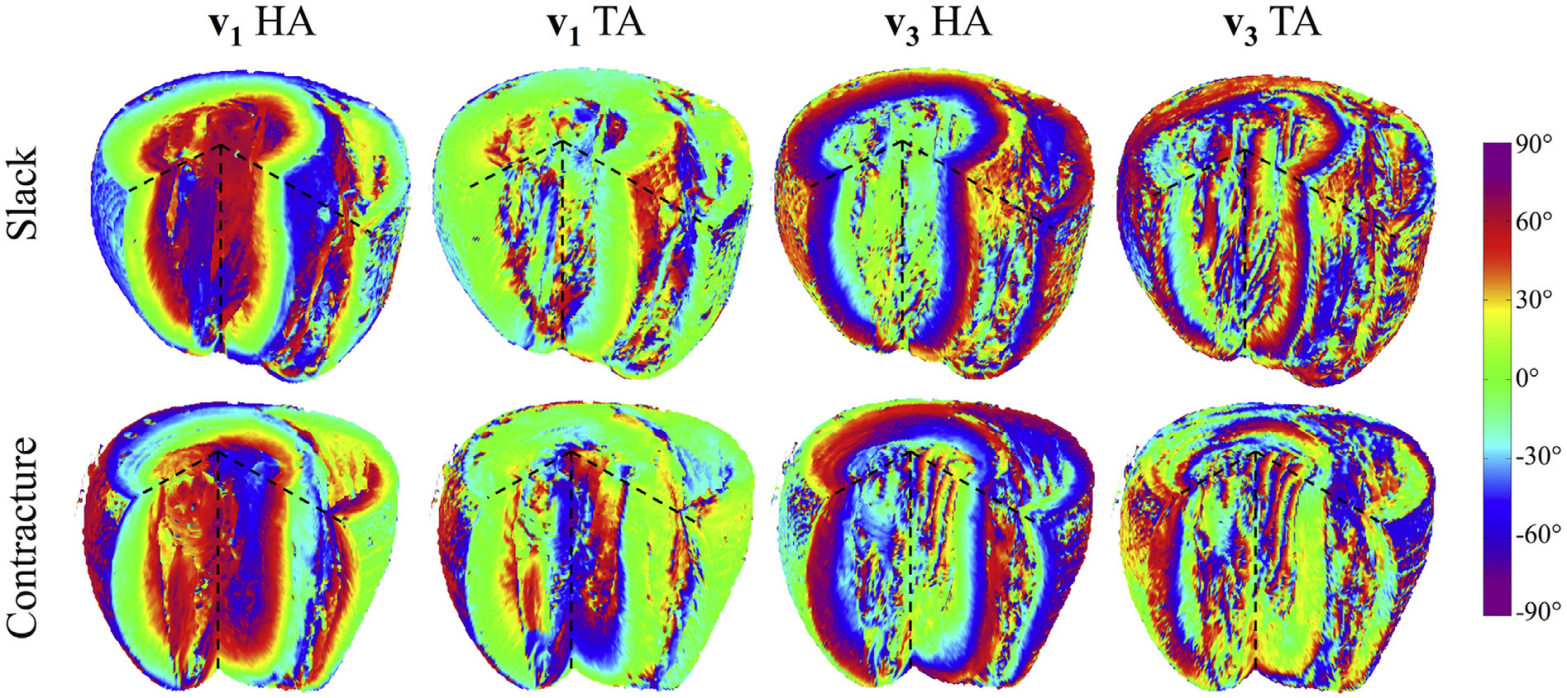
3D distribution of **v**_**1**_ and **v**_**3**_ helix angles (HA) and transverse angles (TA) across a digitally resected myocardium in slack and contractured hearts.

**Fig. 8:**
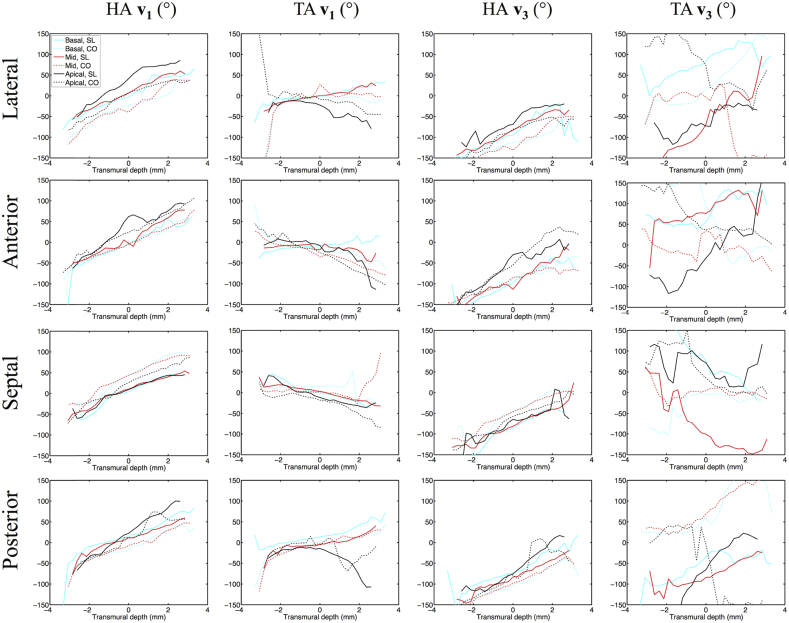
Left ventricular transmural profiles of **v**_**1**_ and **v**_**3**_ helix angles (HA) and transverse angles (TA) across four segments (lateral, anterior, septal and posterior) in three slices (basal, mid and apical) in slack (SL) and contractured (CO) hearts. Profiles are centred on the midline (transmural depth, d = 0 mm) and extend towards the epicardium (d < 0 mm) and endocardium (d > 0 mm).

**Fig. 9:**
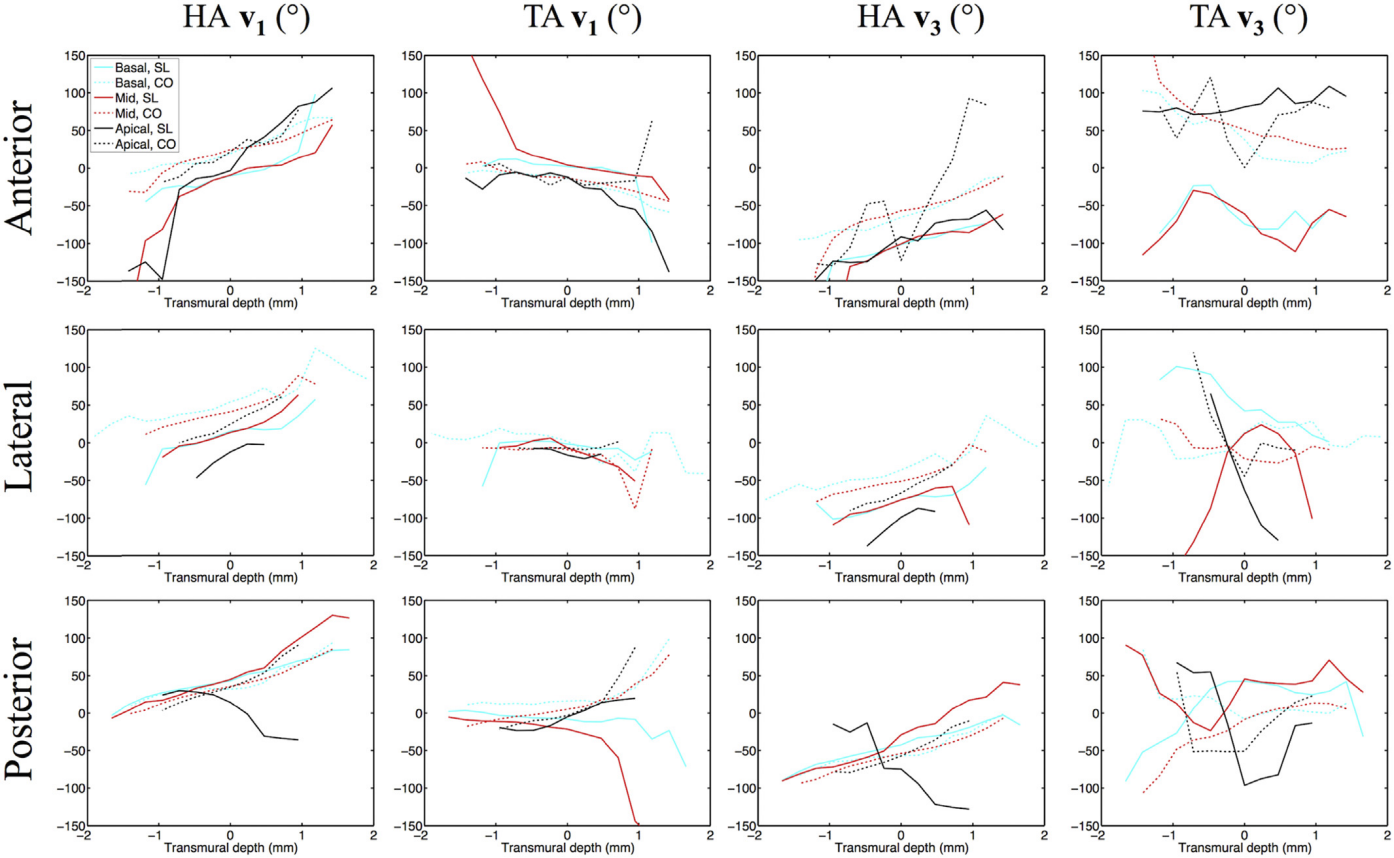
Right ventricular transmural profiles of **v**_**1**_ and **v**_**3**_ helix angles (HA) and transverse angles (TA) across four segments (lateral, anterior, septal and posterior) in three slices (basal, mid and apical) in slack (SL) and contractured (CO) hearts.

**Fig. 10:**
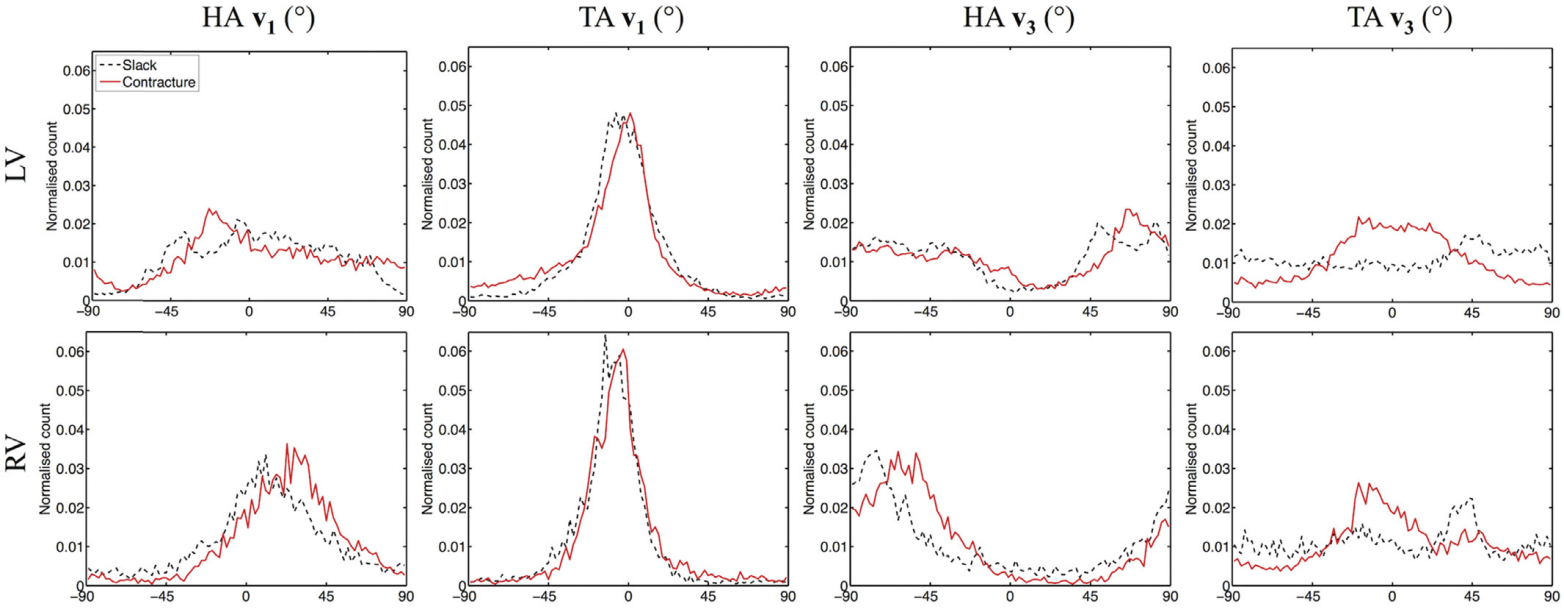
Histograms of **v**_**1**_ and **v**_**3**_ helix angles (HA) and transverse angles (TA) across 3 adjacent mid-ventricular slices in the LV and RV of slack and contractured hearts.

HA (**v**_**1**_, **v**_**3**_) and TA (**v**_**1**_, **v**_**3**_) maps are presented in digitally resected hearts as Fig. 7. Transmural angle profiles were aggregated in several segments of the LV and RV at apical, middle and basal slices and are plotted in Fig. 8, Fig. 9. Linearity and gradient of the profiles in a mid-myocardial slice are summarised in Table 2 and histograms of the angle distributions are plotted in Fig. 10. The transmural variation in HA **v**_**1**_ in the slack heart is relatively uniform and linear across the LV lateral, apical, septal and posterior walls, with gradients in a mid-myocardial slice ranging from 18.2°/mm to 23.6°/mm and linearity ranging from 0.94 to 0.99. In the contractured heart, HA **v**_**1**_ remains linear with R^2^ > 0.94, but there is a constant offset across the myocardium depending on region. For instance, there is a decrease of between 16.3° and 48.5° in the LV lateral endocardium depending on the height of the short-axis slice, and an increase of between 41.7° and 49.9° in the LV septal subendocardium. In the RV of the slack heart, a similar trend of increasingly right helical fibres is observed from the subepicardium to the subendocardium in the anterior, lateral and posterior walls at the middle and basal slices. The gradients in a mid-myocardial slice range from 19.2°/mm to 24.2°/mm depending on region, and are remarkably similar to those in the LV wall of the slack heart. In the RV of the contractured heart, the linear trend towards increasingly right helical fibres in the subendocardium continues with R^2^ > 0.94. However, there is a notable increase in the gradients, which ranges from 28.3°/mm to 31.2°/mm, and a rightward shift of between 25.2° and 34.2° at the midline of the anterior wall, and between 27.1° and 39.0° at the midline of the lateral wall. These descriptions are likewise reflected in the tracking and 3D angle mapping in Fig. 4, Fig. 5, Fig. 6, Fig. 7.

Compared to HA, TA **v**_**1**_ is relatively small in the slack heart, with small gradient values of between −9.2°/mm to 10.7°/mm, signifying a predominance of circumferential orientation in projections of **v**_**1**_ onto the short-axis plane. The majority of the RV is also comprised of largely circumferential fibre with | TA **v**_**1**_ | < 15°. In the contractured heart, TA **v**_**1**_ is similar to that of the slack heart near the midline, but deviates sharply towards the wall surfaces, resulting in an R^2^ of 0.57 compared to 0.79 in the slack hearts, averaged over four regions in a mid-myocardial slice. Most of the fibres in the RV of the contractured heart are also close to circumferential in orientation, with the exception of a region near the RV posterior endocardium where there is a marked increase of 103° compared to slack hearts, averaged over three heights. Sudden large deviations tend to be more prevalent at the LV and RV epicardial and endocardial surfaces. This is a result of the predominantly longitudinal fibre orientation, which greatly reduces the dynamic range of the projection of **v**_**1**_ onto the short-axis plane.

Like HA **v**_**1**_, HA **v**_**3**_ is highly uniform and linear in the LV of the slack heart, with linearity ranging from 0.95 to 0.98 and gradient ranging from 21.1°/mm to 22.8°/mm. **v**_**3**_ is however, by definition orthogonal to **v**_**1**_ and the transition from LV subepicardium to subendocardium is broadly one of right helical to longitudinal to left helical **v**_**3**_. However, this is a simplification of the complex and discrete sheetlet structures that can be seen from Fig. 5. In the contractured heart, linearity of HA **v**_**3**_ is preserved with R^2^ > 0.92, but there is an overall leftward shift of **v**_**3**_ in the LV lateral wall and a rightward shift in the LV septal wall. In the RV in both hearts, there is a similar general trend of right helical through longitudinal to left helical arrangement of **v**_**3**_.

The distributions of TA **v**_**3**_ across the hearts are highly heterogenous, with an average R^2^ of 0.80, 0.10, 0.43 and 0.65 in the LV and RV of the slack heart and the LV and RV of the contractured heart respectively. This is due to the short-range and discrete nature of **v**_**3**_, and the fact that near the midline, **v**_**3**_ is close to longitudinal in orientation. Nonetheless, the histograms in Fig. 10 show that TA **v**_**3**_ has a more circumferential orientation (i.e. smaller |TA **v**_**3**_|) in a sizeable region of the LV and RV in the contractured heart relative to the slack heart.

## 4 Discussion

Our study shows first data on the effects of contracture on DTI parameters in rabbit hearts, and comparisons will be made to studies using other species, with the caveat that there are known structural differences between species (Healy et al., 2011). Here, we found no change in LV and RV FA between slack and contractured hearts. This agrees with findings in a study of isolated rat hearts in multiple states (Hales et al., 2012). In contrast, we found that the mean ADC was lower and the transverse anisotropy was higher in the contractured heart. This result could be attributed to the fact that our *ex vivo* imaging protocol, with its extended acquisition time, afforded higher sensitivity to subtle changes in diffusivity than prior life-heart DTI in rat heart (Hales et al., 2012). On the other hand, that data were acquired in the same heart, while we used separate rabbit hearts for slack and fixed states, with all consequences of inter-individual differences.

Tractography provides a powerful means to identify groups of cells with potential functional relevance. It has been used to visualise conduction pathways to inform electro-mechanical models (Hwang et al., 2011) and to identify structural remodelling in the myocardium post-infarct (Vadakkumpadan et al., 2010). It has also been used to track acceleration fields from phase contrast MRI to estimate LV fibre orientations (Jung et al., 2006) and support structural models of the heart (Buckberg et al., 2006). In this study, we reconstructed extensive fibre tracks across the whole heart based on **v**_**1**_ and **v**_**3**_, summarising information on the 3D fibre and sheetlet architecture. Differences in fibre and sheetlet organisation between the slack and contractured hearts were readily visualised. Localisation of tracks near the apex highlighted dramatic differences in trajectories between slack and contractured hearts, which may inform our understanding of the mechanics of contraction. We observed V-shaped arrangements of sheetlets that are in agreement with MRI (Hales et al., 2012), confocal microscopy (Harrington et al., 2005, Sands et al., 2005) and histological findings (Kung et al., 2011). Evidence was also found of N-shaped sheetlet arrangements and insertion of sheetlets at the intersection of the LV and RV. These observations highlight the complexity in myocardial structure that is still being unravelled. It is worthwhile to keep in mind that the tracks seen are streamlines connecting diffusion tensors and not anatomical features of the heart. The precise organisation of myocardial structure into sheetlets remains a matter of debate. We have no evidence to support the presence of a continuous muscle band, wrapped in 3D to form whole ventricles (Anderson et al., 2005, Torrent-Guasp et al., 2001). However, the continuity of locally prevailing cell orientations, sustained over long distances, is remarkable. This may be an expression of mechanical stress fields, whose presence in the developing heart advance cell maturation (Banerjee et al., 2015) and guide myocardial patterning (Bressan et al., 2014, Nguyen et al., 2015). The view that **v**_**1**_ and **v**_**3**_ reflect myocyte and sheetlet-normal orientations is based on histological validation studies (Holmes et al., 2000, Hsu et al., 1998, Kung et al., 2011, Scollan et al., 1998). However, histology work suffers from tissue distortion and 2D acquisition. Developments in 3D histological reconstruction with methods for correcting tissue distortion promise to further improve the accuracy of these important validation studies (Burton et al., 2014). Besides interpretation, quantification is a challenge in tractography. Parameters such as fibre number and length are sensitive to arbitrary thresholds such as the maximum angle step between adjoining tensors and the minimum FA used for terminating tracks. An initial study investigated how changing thresholds could affect fibre number and length in the heart (Song et al., 2010), but a formal multi-parametric sensitivity analysis is still outstanding. Other technical challenges include intuitive display and interpretation of large numbers of tracks, and questions as to the biological significance of track properties in the heart. In the brain, track number and density have been used to estimate probabilities of connections between grey matter regions (Behrens et al., 2003) and as parameters for inter-subject quantitative analysis (Roberts et al., 2005). Further work is needed to rationalise the utility of track-based statistics in the heart. In any case, fiber and sheetlet tracking are promising techniques that will add a new dimension to conceptual insight and computational modelling of structural and electro-mechanical properties of the heart.

Our measurements of the transmural range of HA **v**_**1**_ in the slack heart were 117° and 120° in the LV lateral and septal walls. This is higher than the 76° and 83° seen in a previous DTI study (Healy et al., 2011), and closer to the values of 131° and 97° reported in histological work (Vetter and McCulloch, 1998). There are a number of reasons that could affect the estimation of **v**_**1**_ in DTI, starting from sample variability and differences in contraction state and fixation. In addition, the resolution of 250 μm used in the earlier study, compared to 200 μm here, could potentially have led to greater voxel averaging and underestimation of absolute helix angles at the epi- and endocardium. Hardware and acquisition parameters such as field strength, maximum gradient strength, and b-value would have affected signal-to-noise ratio and contrast. Differences in post-processing including long-axis calculation, image registration, ROI selection, and transmural profile identification may have also affected the outcome. We observed a well-conserved transmural variation in HA **v**_**1**_. Additionally we found considerable variation in HA and TA throughout the LV and RV. This is not surprising given the 3D angle mapping and tractography results. We also describe HA **v**_**1**_ of the RV and showed that it also varies from left to right helical orientation when progressing form RV epi-to endocardium. It is important to consider that angular measurements are governed by the definition of the local coordinate system. This in turn depends on the nature of the data acquired. Here we acquired 3D isotropic high-resolution data with high SNR and whole heart coverage. This made it possible to accurately segment the hearts and align them to a common global reference frame via rigid registration. Our implementation based on Laplace’s method enabled robust detection of local radial unit vectors in both the LV and RV, in slack and contractured hearts. An alternative approach that has shown promise in in vivo cardiac DTI data with limited slices and SNR, could involve investigating tensor variability using diffeomorphic transformations into prolate spheroidal space (Toussaint et al., 2013).

Measurement of fibre orientation alone is insufficient to inform our understanding of cardiac structure and function. While myocyte contraction provides a basis for force generation and ventricular wall thickening, it does not, for example, fully account for the >25% increase in wall thickness in systole. Instead, changes in sheetlet orientation are thought to increase wall thickness by 16% (Chen et al., 2005), as supported by studies that demonstrated significant transverse shear occurring along the sheetlet planes (Costa et al., 1999, LeGrice et al., 1995). The inclusion of HA and TA data based on **v**_**3**_ shed new light on sheetlet arrangement in the rabbit heart. While the transmural variation in HA **v**_**3**_ was largely linear as per HA **v**_**1**_, it was broadly speaking offset by 90°. In contrast, the distribution of TA **v**_**3**_ was characterised by irregular and large discontinuities. An important consideration is the accuracy in sorting **v**_**2**_ and **v**_**3**_ that depends both on the measurement and sample. Here, mean 95% COU values are low and the precision of **v**_**2**_ and **v**_**3**_ are inversely related to transverse anisotropy, suggesting that the measurement is robust and sorting of **v**_**2**_ and **v**_**3**_ may be problematic in regions with lower tissue orthotropy. These regions include voxels containing substantial isotropic components such as major vessels, and regions with complex cell arrangements such as near the apex. As these regions may be localised, probabilistic (Parker et al., 2003) and global tractography (Reisert et al., 2011) methods could offer improved confidence in tracking over long distances. We observed that transverse anisotropy was higher in the contractured compared to the slack heart, giving rise to better precision in **v**_**2**_ and **v**_**3**_ estimates.

The limitations of the study include a small sample size that precludes statistical analysis, and comparison of two *ex vivo* hearts fixed in different mechanical states. Our prior work using a similar approach yielded consistent results over five *ex vivo* rat hearts, where 95% COU (**v**_**1**_/**v**_**2**_/**v**_**3**_) = 3.7° ± 0.2°/14.7° ± 1.0°/14.4° ± 1.0° (McClymont et al., 2016). This underscores the reproducibility of the DTI measurements, and a larger sample size would improve confidence in sample-related changes in contracture. The second limitation could be resolved by DTI experiments with isolated living hearts observed in multiple mechanical states (Hales et al., 2012), albeit at the cost of signal-to-noise ratio and image resolution. However, fixed tissue *ex vivo* preparations continue to provide data with the highest quality and resolution due to the longer acquisition times and lack of sample motion. In addition, tensors were visualised using superquadric glyphs. While ellipsoids are a more direct representation of the diffusion tensor, superquadric shapes may provide a more intuitive representation of the known orthotropy of myocardial structure.

## 5 Conclusion

Our data reconfirm the highly complex and ordered nature of cardiac architecture. Measuring, understanding and communicating the wealth of macro- and microstructural information is not trivial. To address this challenge, we have combined multiple complementary techniques to quantify and visualise measurements at different length scales. Superquadric glyphs facilitate voxel-wise interrogation of tensors, transmural profiles highlight changes across the myocardial wall, 3D maps describe global parametric distributions, histograms capture summary metrics, and localised and global tractography can potentially identify functionally important groups of fibres and sheetlets.

## Editors’ note

Please see also related communications in this issue by Baumgartner et al. (2016) and Kang et al. (2016).

## Figures and Tables

**Table 1:** Parametric maps in LV and RV of whole hearts: Mean apparent diffusion coefficient (ADC), principal eigenvalues λ_1_, λ_2_, and λ_3_, fractional anisotropy (FA), and transverse anisotropy = λ_2_/λ_3_ (mean ± standard deviation over voxels in whole heart).

		Mean ADC ×10^−3^ mm^2^/s	λ_1_ ×10^−3^ mm^2^/s	λ_2_ ×10^−3^ mm^2^/s	λ_3_ ×10^−3^ mm^2^/s	FA	λ_2_/λ_3_
Slack state	LV	1.34 ± 0.17	1.78 ± 0.16	1.24 ± 0.20	1.02 ± 0.18	0.29 ± 0.06	1.22 ± 0.11
RV	1.45 ± 0.22	1.87 ± 0.26	1.37 ± 0.24	1.10 ± 0.22	0.27 ± 0.07	1.25 ± 0.14
Contractured state	LV	1.25 ± 0.12	1.64 ± 0.14	1.18 ± 0.14	0.93 ± 0.13	0.29 ± 0.05	1.28 ± 0.14
RV	1.40 ± 0.19	1.80 ± 0.23	1.34 ± 0.21	1.05 ± 0.197	0.27 ± 0.07	1.29 ± 0.15

**Table 2:** Linearity and gradient of transmural **v**_**1**_ and **v**_**3**_ helix angle (HA) and transverse angle (TA) profiles in a mid-myocardial slice in lateral (L), anterior (A), septal (S) and posterior (P) LV and RV myocardial walls (Refer to Fig. 1 for segmentation) in a slack and contractured heart.

			**v**_**1**_ HA		**v**_**1**_ TA		**v**_**3**_ HA		**v**_**3**_ TA	
Linearity	Gradient (°/mm)	Linearity	Gradient (°/mm)	Linearity	Gradient (°/mm)	Linearity	Gradient (°/mm)
Slack state	LV	L	0.986	20.5	0.954	10.0	0.973	21.2	0.946	42.9
A	0.971	23.6	0.430	−4.0	0.945	22.8	0.622	17.9
S	0.967	18.3	0.943	−9.2	0.950	21.4	0.825	−33.4
P	0.941	18.2	0.817	10.7	0.975	21.1	0.792	16.3
RV	A	0.766	64.0	0.757	−55.6	0.760	74.5	0.005	2.0
L	0.946	37.7	0.701	−23.5	0.199	13.3	0.302	59.9
P	0.964	41.4	0.751	−55.0	0.977	42.2	0.000	0.5
Contractured state	LV	L	0.963	24.2	0.404	17.5	0.958	24.9	0.475	−25.6
A	0.987	19.2	0.971	−13.9	0.923	13.7	0.267	−6.8
S	0.973	22.0	0.113	4.0	0.973	21.8	0.102	−3.2
P	0.941	19.6	0.785	15.2	0.942	24.0	0.879	27.7
RV	A	0.947	31.2	0.963	−16.6	0.607	62.4	0.681	−47.2
L	0.942	29.8	0.283	−16.3	0.922	28.7	0.438	−15.7
P	0.983	28.3	0.867	28.0	0.983	27.6	0.837	38.0

## List of abbreviations

2D: two-dimensional
3D: three-dimensional
ADC: apparent diffusion coefficient
DTI: diffusion tensor imaging
FA: fractional anisotropy
HA: helix angle
MRI: magnetic resonance imaging
ROI: region-of-interest
TA: transverse angle
